# Extreme bioengineering to meet the nitrogen challenge

**DOI:** 10.1073/pnas.1812247115

**Published:** 2018-08-16

**Authors:** Stefan Burén, Gema López-Torrejón, Luis M. Rubio

**Affiliations:** ^a^Centro de Biotecnología y Genómica de Plantas, Universidad Politécnica de Madrid, Instituto Nacional de Investigación y Tecnología Agraria y Alimentaria, Pozuelo de Alarcón, 28223 Madrid, Spain;; ^b^Departamento de Biotecnología-Biología Vegetal, Escuela Técnica Superior de Ingeniería Agronómica, Alimentaría y de Biosistemas, Universidad Politécnica de Madrid, 28040 Madrid, Spain

In the 1970s, investigators were already envisioning new technologies to generate crops that could utilize nitrogen (N_2_) from air to produce their own fixed N species, stating that “cereals that could provide their own fertilizer are beyond doubt the biggest prize of all in the gift of the new biology” (1). At this time, the N_2_-fixation genes (*nif*) of *Klebsiella pneumoniae* had just been transferred to *Escherichia coli*, making the naturally nonfixing bacterium capable of growing in the absence of combined N (2) and giving hope that this feature could also be carried over to more complex organisms. In PNAS, almost 50 years after the generation of the first N_2_-fixing *E. coli*, researchers from the Peking University, in collaboration with the lead author from that breakthrough study, present an ingenious approach to achieve coordinated and stoichiometric expression of *nif* genes in heterologous hosts (3). The method is based on regrouping genes of complex biological pathways into synthetic “giant genes,” products of which may be further processed into shorter functional proteins on demand. This technology will revolutionize our attempts to transfer the elaborate *nif* machinery into heterologous hosts, providing researchers with a groundbreaking advance to help generate plants capable of self-fertilization (4, 5).

Successful transfer of *nif* genes into nonfixing organisms, such as plants, will depend on our ability to execute rational simplifications. Researchers must aim at finding the least possible complex, and most possible robust, genetic components capable of performing nitrogenase-related reactions in cellular environments different from their natural hosts. We must also identify intermediate steps of nitrogenase biosynthesis and develop methods for their analysis so that nitrogenase can be stepwise engineered—and functionally verified—before integration into a complete pathway. Once these objectives are achieved, synthetic biology, combined with increased knowledge of nitrogenase biogenesis and catalysis, will enable fine-tuning and improvement of complete N_2_-fixing reaction.

Reasons for the need of simplification are plentiful. Genes that produce soluble, stable, and functional proteins in eukaryotic target hosts must be identified. Expression and regulation of multiple *nif* genes must be controlled, as they are naturally tightly regulated (6, 7). Functionality of some Nif components depend on the correct formation of multimeric complexes, such as NifDK and NifEN; others form more transient interactions that assist in Nif protein folding, protection, and delivery of cofactors or electrons (8). Finally, nitrogenase functionality depends on the biophysical environment of the new cellular host. Well-known factors affecting nitrogenase include sensitivity of its metal clusters to oxygen exposure, ATP levels and reducing power required for the nitrogenase reaction, and metal availability and homeostasis for the biosynthesis of the various nitrogenase cofactors (9, 10).

Reducing nitrogenase genetic requirements to a minimum will likely be crucial for transfer to eukaryotic hosts. Current advances in synthetic biology have provided instruments to pursue the ideas put forward in the 1970s, and recent studies have shown how these new tools can rewrite and refactor naturally occurring *nif* gene clusters. In excellent studies involving *K. pneumoniae* or *Klebsiella oxytoca* as *nif* sources, gene clusters were reorganized or refactored and transferred to *E. coli*. Nonessential genetic parts could be cleaned up, and native *nif* regulation was replaced with fully defined expression units that enabled analysis and optimization of the heterologous *E. coli* nitrogenase (11–14).

In another study published in PNAS last year by these authors (15), synthetic biology was employed to reconstruct electron-transport components consisting of ferredoxin-NADPH oxidoreductases and ferredoxins originating from plant organelles. Their ability to feed electrons to nitrogenase was tested to identify plant or plant-related proteins that could perform some of the reactions needed for nitrogenase to reduce the number of prokaryotic genes needed to generate a putative N_2_-fixing plant.

In recent years, we have also seen attempts to express nitrogenase components in eukaryotic cells (e.g., *Saccharomyces cerevisiae* and *Nicotiana benthamiana*), and in vitro activity of purified yeast NifH and NifB proteins were reported (16–19). NifH was only active upon targeting to the mitochondria when cultured cells were grown under aerobic conditions, strengthening the assumption that ATP production and O_2_ consumption will be important for the functionality of plant nitrogenase (20). Unfortunately, while mitochondrial targeting minimizes O_2_ exposure, it introduces another challenge, as the proteins now have to be equipped with a leader sequence that must be properly cleaved upon mitochondrial import. In the perhaps most elaborate eukaryotic Nif study published so far, nine *nif* genes (*nifHDKUSMENB*) were cotransferred to the genome of yeast (21). In contrast to the other two studies that used strong and inducible galactose-dependent promoters to drive Nif expression, constitutive promoters were designed here to render expression levels and stoichiometries more similar to the natural *nif* system (6). From this work, it became clear that accumulation of Nif proteins in mitochondria did not always correlate to expected expression levels and that mitochondria leader sequences could influence Nif protein accumulation. Importantly, expression of NifDK and NifEN heterotetramers proved difficult, indicating that complex formation upon import to the mitochondria was challenging.

To reduce the number of genes transferred and to enable stoichiometric expression of Nif components in eukaryotic cells, internal ribosome entry site (IRES) technology or so-called 2A peptides can be employed to produce multiple proteins from single transcripts. While IRES initiates translation in a cap-independent manner, the 2A peptide impairs formation of the peptide bond. As translation continues, it results in “ribosomal skipping” or “cleavage.” The 2A peptide provides expression at equimolar levels, but it also leaves an approximately 20-amino acid extension at the carboxyl terminus of the protein upstream of the 2A peptide and an extra proline residue at the amino terminus of the protein downstream. These extensions can impair functionality or stability of proteins. While 2A peptides and IRES have successfully been used in mammalian cells—for example, for the creation of multicistronic gene-expression cassettes capable of generating induced pluripotent stem cells (22)—targeting and cleavage efficiency in plants have been questioned (23).

In the PNAS article, Yang et al. (3) provide an ingenious methodology that could overcome many of the above-listed hurdles. Instead of using IRES or 2A peptides, the authors exploit a posttranslational protein splicing strategy based on the tobacco etch virus protease (TEVp). TEVp specifically cleaves polypeptides harboring the ENLYFQS sequence (at Q\S), leaving shorter overhangs (6 and 1 amino acids at the carboxyl and amino termini, respectively) than 2A peptides and enabling multiple genes to be fused and expressed as giant polypeptides that subsequently generate individual proteins by the action of TEVp. First, Yang et al. (3) analyzed expression levels of 18 *nif* genes endowing *E. coli* with N_2_ fixation to assign them to subgroups. Then, the “tailing tolerance” was determined for each Nif protein. Taking into consideration the native expression levels and tailing tolerance and further eliminating four *nif* genes that proved to be nonessential for *E. coli* (*nifTXZQ*), five giant genes were constructed (*nifHǒDǒK*, *nifEǒN∼B*, *nifUǒS*, *nifJǒVǒW*, and *nifFǒMǒY*). Expression of these giant genes in *E. coli* resulted in 72% of nitrogenase activity relative to the original system and supported diazotrophic growth of the bacterium. Quantitative analysis of individual protein levels revealed that stoichiometry of most components from this polyprotein-based expression system correlated well with the reconstituted operon-based system in *E. coli* and with the native system in the original *K. oxytoca* host.

**Fig. 1.**
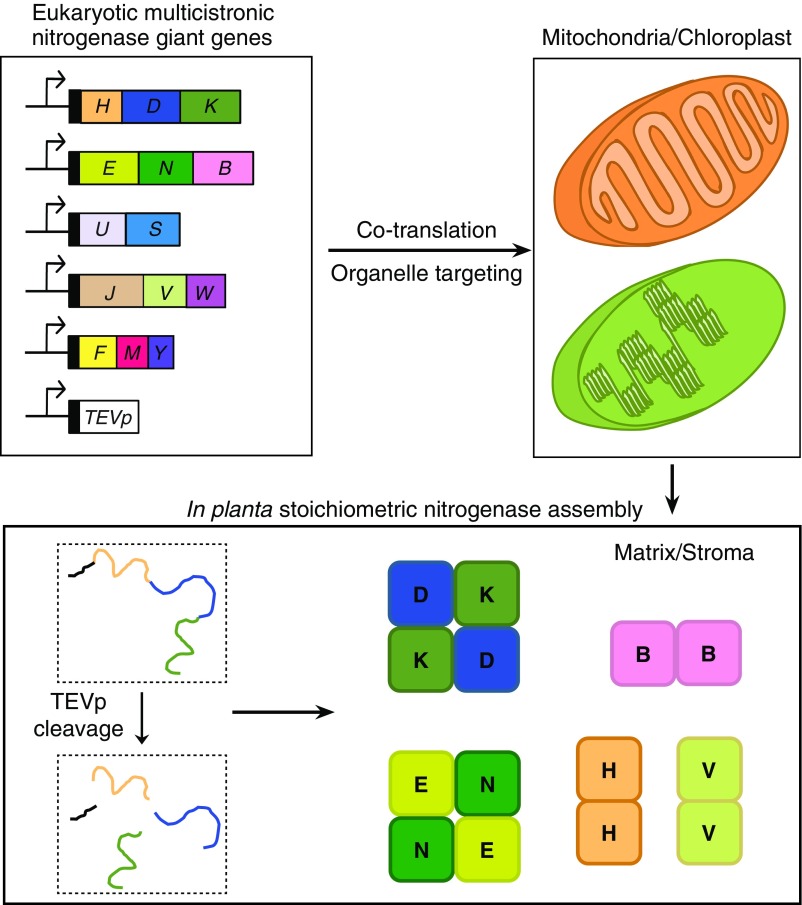
Prospects for giant genes in plant nitrogenase engineering.

The Yang et al. work (3) provides a conceptual advance to generate N_2_-fixing plants by describing a method for (Nif) protein synthesis from multicistronic genes with much shorter carboxyl-terminal overhang than would be the case from using 2A peptides (Fig. 1). While this reduces the tailing effect, even more important is that it facilitates organelle targeting, as would be the case if nitrogenase were to be located at the plant mitochondrial matrix. Because both IRES and 2A peptide results in “cleavage” at the site of translation (cytoplasm), subsequent mitochondria/chloroplast targeting would require targeting signals for each and every cleaved polypeptide. However, using TEVp, only one leader sequence will be needed per giant gene, facilitating cloning in plant expression vectors and transformation of plant genomes. In addition, because cleavage would occur within the organelle itself (where TEVp is targeted), folding of multimeric Nif proteins (e.g., NifDK and NifEN) will likely be improved. This technology could also help taking advantage of previous bacterial *nif* refactoring work (11–14) by creating eukaryotic “operons” that minimize the number of required transcriptional units, thus making *nif* gene expression more robust and easier to optimize. It will be crucial to test how well it performs in eukaryotic cells and importantly upon organelle targeting. TEVp must not be active outside the organelle because it would prevent import of the proteins downstream of the primary TEVp recognition site. If successful in eukaryotic organelles, this technology will be a milestone in the path to N_2_-fixing plants.
